# Cryo-Electron Microscopy Structures of a Campylobacter Multidrug Efflux Pump Reveal a Novel Mechanism of Drug Recognition and Resistance

**DOI:** 10.1128/spectrum.01197-23

**Published:** 2023-06-08

**Authors:** Zhemin Zhang, Nicholas Lizer, Zuowei Wu, Christopher E. Morgan, Yuqi Yan, Qijing Zhang, Edward W. Yu

**Affiliations:** a Department of Pharmacology, Case Western Reserve University School of Medicine, Cleveland, Ohio, USA; b Department of Veterinary Microbiology, College of Veterinary Medicine, Iowa State University, Ames, Iowa, USA; University of Hong Kong

**Keywords:** *Campylobacter jejuni*, cryo-EM, multidrug efflux pump, multidrug resistance, RE-CmeB

## Abstract

Campylobacter jejuni is a bacterium that is commonly present in the intestinal tracts of animals. It is also a major foodborne pathogen that causes gastroenteritis in humans. The most predominant and clinically important multidrug efflux system in C. jejuni is the CmeABC (Campylobacter multidrug efflux) pump, a tripartite system that includes an inner membrane transporter (CmeB), a periplasmic fusion protein (CmeA), and an outer membrane channel protein (CmeC). This efflux protein machinery mediates resistance to a number of structurally diverse antimicrobial agents. A recently identified CmeB variant, termed resistance enhancing CmeB (RE-CmeB), can increase its multidrug efflux pump activity, likely by influencing antimicrobial recognition and extrusion. Here, we report structures of RE-CmeB in its apo form as well as in the presence of four different drugs by using single-particle cryo-electron microscopy (cryo-EM). Coupled with mutagenesis and functional studies, this structural information allows us to identify critical amino acids that are important for drug resistance. We also report that RE-CmeB utilizes a somewhat unique subset of residues to bind different drugs, thereby optimizing its ability to accommodate different compounds with distinct scaffolds. These findings provide insights into the structure-function relationship of this newly emerged antibiotic efflux transporter variant in Campylobacter.

**IMPORTANCE** Campylobacter jejuni has emerged as one of the most problematic and highly antibiotic-resistant pathogens, worldwide. The Centers for Disease Control and Prevention have designated antibiotic-resistant C. jejuni as a serious antibiotic resistance threat in the United States. We recently identified a C. jejuni resistance enhancing CmeB (RE-CmeB) variant that can increase its multidrug efflux pump activity and confers an exceedingly high-level of resistance to fluoroquinolones. Here, we report the cryo-EM structures of this prevalent and clinically important C. jejuni RE-CmeB multidrug efflux pump in both the absence and presence of four antibiotics. These structures allow us to understand the action mechanism for multidrug recognition in this pump. Our studies will ultimately inform an era in structure-guided drug design to combat multidrug resistance in these Gram-negative pathogens.

## INTRODUCTION

Campylobacter jejuni is a major foodborne pathogen that causes gastroenteritis in humans and accounts for >400 million cases of diarrhea each year worldwide (1, 2). The transmission of infection to humans is primarily via the consumption of contaminated foods, such as undercooked poultry and raw milk, as C. jejuni is widely present in the intestinal tracts of most animals. Enteritis is the primary clinical manifestation of campylobacteriosis (3), but extraintestinal infections, such as bacteremia (4, 5), hepatitis (6), and pancreatitis (7), have been occasionally reported. Additionally, postinfection complications, including reactive arthritis (8) and neurological disorders, such as Guillain-Barré syndrome, which is a polio-like form of paralysis (2), have been documented. Antibiotic therapy is administered for prolonged and severe infections, but Campylobacter has unfortunately developed resistance to multiple antimicrobials, mainly due to the frequent use and overuse of antibiotics in animal agriculture and medical settings (9–11). Due to the rising prevalence of antibiotic-resistant Campylobacter and its negative impact on public health, the Centers for Disease Control and Prevention (CDC) have designated antibiotic-resistant Campylobacter as a serious antibiotic resistance threat in the United States (12, 13).

Multidrug efflux is a powerful antibiotic resistance mechanism in bacteria and a major cause of the failure of drug-based treatments in infectious diseases (14). In C. jejuni, the best characterized and most functionally important efflux system that mediates multidrug resistance (MDR) to structurally diverse antimicrobials is the Cme (Campylobacter multidrug efflux) tripartite system (15–17). The Cme locus consists of three tandemly linked genes, namely, *cmeABC*, which encodes the CmeA, CmeB, and CmeC protein components of this tripartite efflux system, in which all three components are required for substrate expulsion. This tripartite system contains the CmeB inner membrane multidrug efflux pump which belongs to the resistance-nodulation-cell division (RND) superfamily of transport proteins (18). CmeB constitutes both the substrate binding sites and the proton-relay network that generates the proton motive force (PMF). CmeB works with the CmeA periplasmic membrane fusion protein and the CmeC outer membrane channel to actively export antimicrobials out of bacterial cells (15, 19).

We originally identified a C. jejuni genetic variant of *cmeABC* in the Campylobacter coli isolate DH161 (20). This variant is significantly more potent than the typical wild-type *cmeAB*C against multiple antibiotics, conferring an exceedingly high level of resistance to fluoroquinolones in conjunction with mutations in the DNA gyrase subunit A (20). C. jejuni carrying the variant *cmeABC* gene was also found to expand the mutant selection window of ciprofloxacin, enhance the emergence of spontaneous ciprofloxacin-resistant mutants, and shift the MIC distribution of various antibiotics to a higher range among clinical C. jejuni isolates. Therefore, we designated this CmeABC variant as RE-CmeABC (resistance enhancing CmeABC) (20). RE-CmeABC represents an emerging multidrug resistance mechanism and an effective strategy that is utilized by Campylobacter for adaptation to antibiotic selection pressure. Using gene replacement, we discovered that amino acid changes in the CmeABC tripartite efflux system were associated with elevated levels of multidrug resistance (20). Additionally, computer-based molecular modeling suggested that amino acid substitutions in the drug-binding pocket of RE-CmeB might contribute to its enhanced function in multidrug resistance (20). These findings demonstrate a sequence variation-mediated mechanism that significantly enhances the function of multidrug resistance pumps. However, the detailed molecular mechanisms and the structural basis for the enhanced multidrug resistance function remain to be determined.

To elucidate how RE-CmeB elevates drug resistance and enhances transport function, we determined the cryo-EM structures of this membrane protein. Here, we present the cryo-EM structures of RE-CmeB, both in the absence and presence of the antibiotics ciprofloxacin (Cip), chloramphenicol (Chl), erythromycin (Ery), and hydrolyzed ampicillin (Amp) to resolutions between 3.08 Å and 3.39 Å. Based on these structures, we identified important drug-binding residues and modes of RE-CmeB-drug interactions. The Cip molecule is observed to bind mainly at the distal-drug binding site in the periplasmic domain of the pump. However, the bound Chl, Ery, and Amp drugs are found to span to both the proximal and distal drug-binding sites. These four substrate binding sites partially overlap each other. It appears that the RE-CmeB efflux pump utilizes slightly different subsets of amino acids to bind these drugs, thereby optimizing the capacity of recognizing and effectively extruding a broad spectrum of distinct classes of antibiotics.

## RESULTS

### Structures of RE-CmeB in the absence of added drugs.

We recently determined the X-ray structure of C. jejuni CmeB (strain NCTC 11168) (21). We also used single-molecule fluorescence resonance energy transfer (sm-FRET) imaging to elucidate the functional dynamics of this multidrug efflux pump (21). We found that CmeB assembles as a trimer and that individual protomers of CmeB function independently to transport drugs within the trimer (21). C. jejuni RE-CmeB and the typical wild-type CmeB (C. jejuni strain NCTC 11168) multidrug efflux pumps share 81% protein sequence identity. To elucidate the structure of C. jejuni RE-CmeB and to understand how this pump elevates the level of resistance to antibiotics, we expressed recombinant C. jejuni RE-CmeB by cloning the *RE-cmeB* sequence into the Escherichia coli expression vector pET15b with a 6×His tag at the N terminus to generate pET15bΩ*cmeB_RE_*. This RE-CmeB protein was overproduced in E. coli BL21(DE3)Δ*acrB* cells and was purified using a Ni^2+^-affinity column. We reconstituted purified RE-CmeB into lipidic nanodiscs and collected single-particle images of this pump using cryo-electron microscopy (cryo-EM). The extensive classification of the single-particle images indicated that two distinct populations of RE-CmeB coexisted in this nanodisc sample. Several iterative rounds of classifications allowed us to sort the images based on these two distinct classes. One of these structures depicted that the trimeric RE-CmeB pump does not contain any bound ligands. In the second RE-CmeB structure, it was observed that one of the RE-CmeB protomers within the trimer possesses a bound ligand.

**FIG 1:**
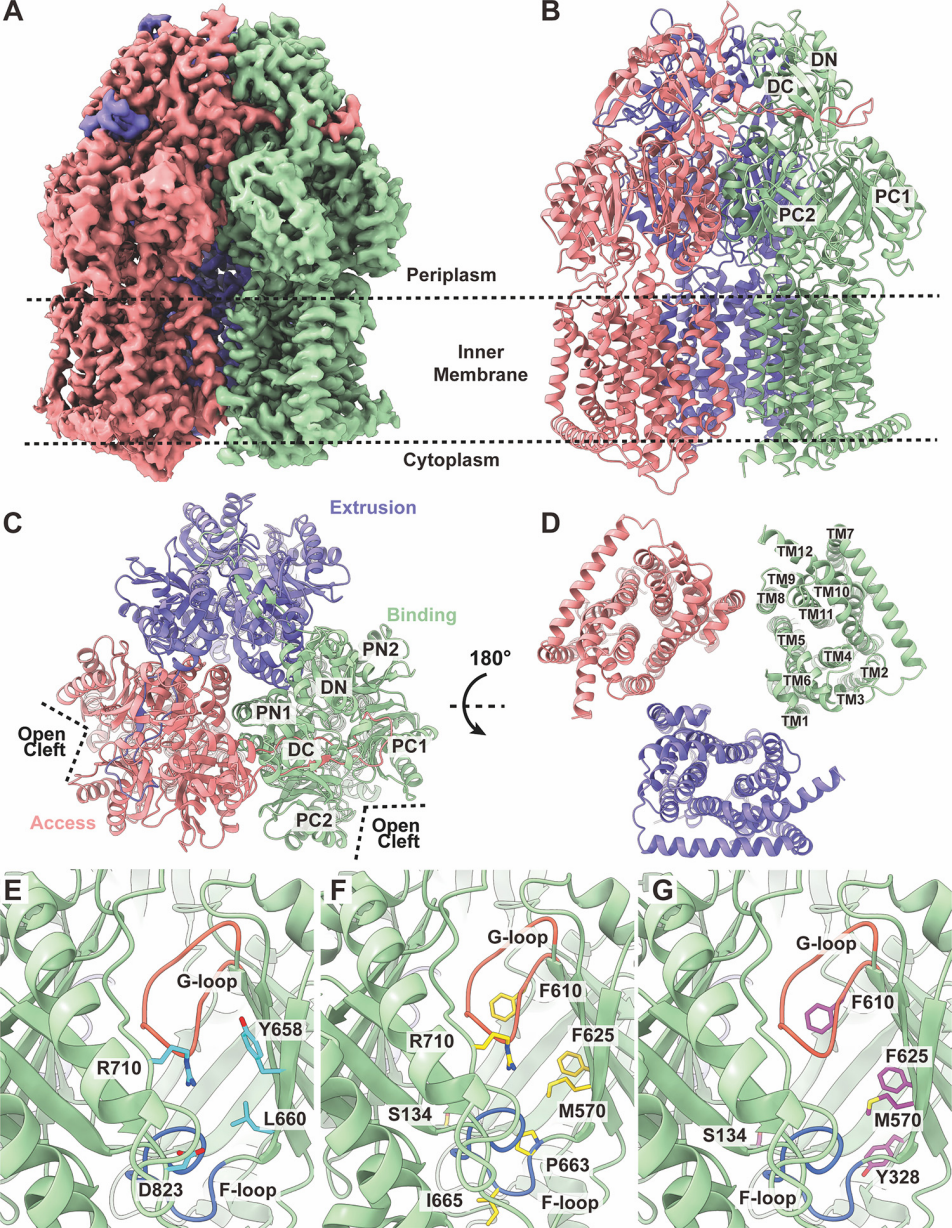
The cryo-EM structure of C. jejuni RE-CmeB. (A) Cryo-EM density map of trimeric RE-CmeB. (B–D) Ribbon diagrams of the structure of RE-CmeB (panel B, side view; panel C, top view; panel D, bottom view). In panels B–D, the “access”, “extrusion”, and “binding” protomers are colored pink, green, and slate, respectively. Each protomer of RE-CmeB contains 12 transmembrane helices (TM1-TM12) and 6 periplasmic subdomains (PN1, PN2, PC1, PC2, DN, and DC). (E) The entrance drug-binding site. The residues that are predicted to contribute to form the entrance drug-binding site are highlighted with cyan sticks. (F) The proximal drug-binding site. The residues that are predicted to contribute to form the proximal drug-binding site are highlighted with yellow sticks. (G) The distal drug-binding site. The residues that are predicted to contribute to form the distal drug-binding site are highlighted with purple sticks. In (E–G), the secondary structural elements of the “binding” protomer of RE-CmeB are colored green. The F- and G-loops are colored blue and orange.

**(i) Structure of apo-RE-CmeB.** The cryo-EM images of RE-CmeB without a bound ligand permitted us to resolve the structure of apo-RE-CmeB at a nominal resolution of 3.08 Å (Fig. 1; Fig. S1; Table S1). This multidrug efflux pump adopts the overall architecture of RND-type proteins and forms a homotrimer (21–29). Each protomer is folded into a transmembrane domain containing 12 transmembrane helices (TM1 to TM12) and a periplasmic domain that can be divided into six subdomains (PN1, PN2, PC1, PC2, DN, and DC) (Fig. 1A–D). Subdomains PC1 and PC2 create a periplasmic cleft that allows substrates to enter the pump via the periplasm. Deep inside the cleft, each protomer of RE-CmeB creates multiple substrate-binding pockets, including the proximal and distal multidrug-binding sites. Substrates entering the periplasmic cleft are likely sequentially bound by the entrance, proximal, and distal sites for substrate extrusion.

The entrance of the periplasmic cleft of RE-CmeB is encompassed by the amino acids Y658, L660, R710, and D823 (Fig. 1E). These residues appear important for the specificity and selectivity of the RE-CmeB substrates. Among these four residues, only R710 is conserved between RE-CmeB and wild-type CmeB. Interestingly, R710 is also a conserved residue among C. jejuni RE-CmeB, E. coli AcrB (22), N. gonorrhoeae MtrD_CR103_ (30, 31), P. aeruginosa MexB (24), A. baumannii AdeB (26, 32), and A. baumannii AdeJ (33, 34) pumps, with the corresponding arginines R717 of AcrB (35), R716 of MexB (36), and R714 of MtrD_CR103_ (30) being found to be critical for the function of these pumps.

A flexible F-loop (^663^PPIPGLSL^670^) is observed to connect the periplasmic entrance and the proximal multidrug-binding sites of RE-CmeB. Some of these F-loop residues also structurally contribute to the floor of the proximal multidrug-binding site.

At least 22 amino acids have been reported to contribute to the formation of the proximal drug-binding site of AcrB (37). Among them, 14 of these residues are conserved with those of wild-type CmeB. However, of these 22 residues, only 7 are highly conserved among the RE-CmeB, AcrB, MtrD_CR103_, AdeB, and AdeJ pumps (22, 26, 30, 32–34). These RE-CmeB residues are S134, M570, F610, F625, P663, I665, and R710 (Fig. 1F).

The gate G-loop of RE-CmeB is generated by the loop ^609^GFDLFTSSLKEN^620^, where G609, F610, and N620 are conserved among RE-CmeB, AcrB, MtrD_CR103_, AdeB, and AdeJ. Molecular dynamics simulations suggest that the G-loop phenylalanines of the AcrB pump are important for the process of transfer and stabilizing substrate binding (37). The G-loop phenylalanines have been observed to engage in binding substrates in AdeB (32), MtrD_CR103_ (30, 31), and AcrB (38). Interestingly, the G-loop of RE-CmeB is two amino-acids longer compared with those of AcrB (22), MtrD_CR103_ (30, 31), AdeB (26, 32), and AdeJ (33, 34). This extended RE-CmeB G-loop may impact the binding of drugs, as it may allow for more flexibility to help accommodate pump-drug interactions.

Similar to the proximal multidrug-binding site, the number of residues that comprise the distal multidrug-binding site is extensive. At least 23 amino acids are involved in forming the distal site of AcrB (23, 37). Among these residues, five of them are highly conserved in the RE-CmeB, AcrB (22), MtrD_CR103_ (30, 31), AdeB (26, 32), and AdeJ (33, 34) pumps. The corresponding RE-CmeB residues are S134, Y328, M570, F610, and F625 (Fig. 1G). In AcrB, a hydrophobic patch that is found at the ceiling of the distal drug-binding site has a strong impact on pump-drug interactions (37). A similar hydrophobic patch is found in RE-CmeB, and this RE-CmeB hydrophobic patch is composed of I178, L607, and F610. Similar to AcrB, these patch residues may also be important for drug binding. It should be noted that among the 23 residues that form the distal drug-binding site of RE-CmeB, only 11 residues are conserved with those of wild-type CmeB.

Interestingly, the cryo-EM structure of RE-CmeB depicts that this multidrug efflux pump forms an asymmetric trimer in which the three protomers display distinct conformational states. Based on the X-ray structures of wild-type CmeB from C. jejuni strain NCTC 11168, we found that the trimeric CmeB pump can assemble as both symmetric and asymmetric trimers. Each protomer of CmeB in the symmetric trimer displays a “resting” conformational state. However, the three protomers of the CmeB asymmetric trimer depict the “binding”, “extrusion”, and “resting” conformations (21). The superimposition of the cryo-EM structure of trimeric RE-CmeB onto the X-ray structure of the asymmetric CmeB trimer gives rise to an RMSD of 1.7 Å, indicating that the structures of these two asymmetric trimers are somewhat distinct from each other. Similar to the structures of asymmetric AcrB (23, 39), MtrD_CR103_ (30, 31), AdeB (26, 32), and AdeJ (33, 34), the three RE-CmeB protomers in our cryo-EM structure present the “access”, “binding”, and “extrusion” conformations, respectively (Fig. 1A–C; Table S2).

**FIG 2:**
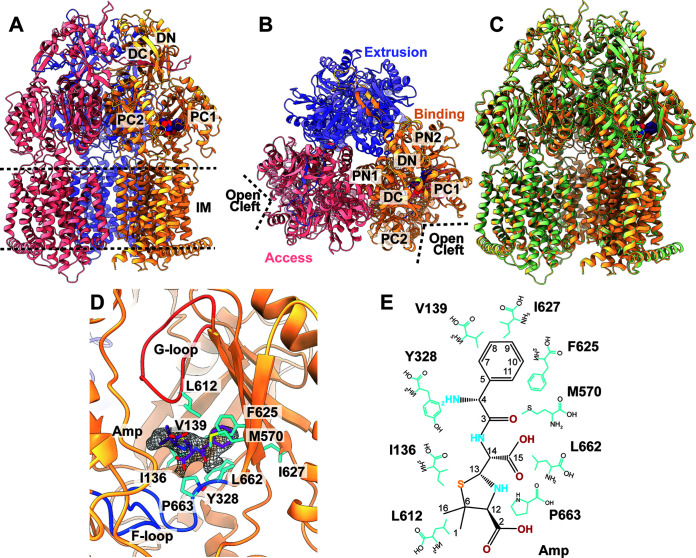
Cryo-EM structure of RE-CmeB-Amp. (A) Ribbon diagram of the structure of the side view of the RE-CmeB-Amp trimer. (B) Ribbon diagram of the structure of the top view of the RE-CmeB-Amp trimer. In panels A and B, the “access”, “binding”, and “extrusion” state protomers are colored in pink, slate, and orange, respectively. (C) The superimposition of the structures of apo-RE-CmeB and RE-CmeB-Amp. The secondary structural elements of apo-RE-CmeB and RE-CmeB-Amp are colored green and orange, respectively. This superimposition gives rise to an RMSD of 0.6 Å. In panels A–C, the bound Amp molecule within the “binding” protomer of RE-CmeB-Amp is colored purple. (D) The Amp binding site. The residues that participate in Amp binding are shown with cyan sticks. The bound Amp molecule is colored purple. The cryo-EM density of Amp is colored gray. The F-loop and G-loop are colored blue and orange, respectively. (E) The schematic diagram of the Amp binding site. The amino acids that are important for Amp binding are shown as cyan sticks.

**(ii) Structure of RE-CmeB-Amp.** In addition to the apo-RE-CmeB structure, we also observed a separate distinct class of images of RE-CmeB from our cryo-EM data. These images led to a high-quality cryo-EM map (Fig. 2; Fig. S1; Table S1), thereby allowing us to solve the structure of this RE-CmeB-Amp complex at a resolution of 3.16 Å. Like apo-RE-CmeB, the structure of RE-CmeB-Amp presents an asymmetric trimer in which the three RE-CmeB protomers can be easily classified as the “access”, “binding”, and “extrusion” conformers, respectively (Fig. 2A and B; Table S2). The superimposition of the apo-RE-CmeB and RE-CmeB-Amp structures shows that these two trimers are similar, with an overall root-mean-square-deviation (RMSD) of 0.6 Å (Fig. 2C). Different from apo-RE-CmeB, an extra density was found within the periplasmic multidrug binding cavity of the “binding” protomer of RE-CmeB, indicating that this multidrug efflux pump is bound by a fortuitous ligand. However, no extra densities were found within the periplasmic drug-binding sites of the “access” and “extrusion” protomers. The shape of this extra density is compatible with a hydrolyzed ampicillin (Amp) antibiotic with an open form of the four-member β-lactam ring (Fig. 2D and E). This is unexpected but not surprising, as we supplemented Luria-Bertani (LB) broth with 100 μg/mL ampicillin to grow E. coli BL21(DE3)Δ*acrB* cells that harbor the pET15bΩ*cmeB_RE_* plasmid to express the RE-CmeB membrane protein. The ampicillin molecules were probably hydrolyzed in the LB solution during cell growth at 37°C. Interestingly, this bound Amp molecule was also found in the cryo-EM structure of the Neisseria gonorrhoeae MtrD_CR103_ multidrug efflux pump that was previously determined from our lab (30). Like RE-CmeB, this MtrD_CR103_ pump can enhance the pump’s activity and further elevate the resistance levels to multiple antimicrobials. However, the mode of Amp binding from RE-CmeB is different from that of MtrD_CR103_. It is observed that the bound Amp drug is located directly below the G-loop, where it spans both the periplasmic proximal and distal drug binding sites of the “binding” protomer of RE-CmeB. Within 4 Å of bound Amp, the RE-CmeB residues I136, V139, Y328, M570, L612, F625, I627, L662, and P663 are engaged in anchoring this deactivated drug (Fig. 2D and E). Notably, all of these residues are hydrophobic in nature, presumably providing hydrophobic interactions at this distal site to stabilize substrate binding.

### Structure of the RE-CmeB in the presence of antibiotics.

Since the 1980s, fluoroquinolones have been a key class of antibiotics used for treating Campylobacter infections in humans (3). However, fluoroquinolone-resistant Campylobacter is increasingly prevalent, which has limited the use of fluoroquinolones as therapeutic drugs in patients. Currently, macrolides are the drugs of choice for the antibiotic treatment of Campylobacter infections (3). To elucidate how RE-CmeB recognizes fluoroquinolone and macrolide antibiotics, we defined the cryo-EM structures of the RE-CmeB-Cip and RE-CmeB-Ery complexes. We also solved the cryo-EM structure of the RE-CmeB-Chl complex, as RE-CmeB can mediate a high level of resistance to this antibiotic. The structures illustrate that these three drugs bind to the “binding” protomer of RE-CmeB. However, these drugs are anchored to various positions, yet they partially overlap. The Cip molecule is mostly bound at the distal drug binding site, but Ery and Chl span both the proximal and distal sites. The binding mode of spanning two multidrug-binding sites is novel and is distinct from those observed in AcrB (23, 38), MtrD_CR103_ (30), and AdeJ (33, 34). It has also been observed that each drug uses a slightly different subset of RE-CmeB residues to secure the binding, thereby greatly potentiating the range of drug-pump interactions. Below we describe the structural basis of pump-drug interactions in these three RE-CmeB-ligand complexes.

**FIG 3:**
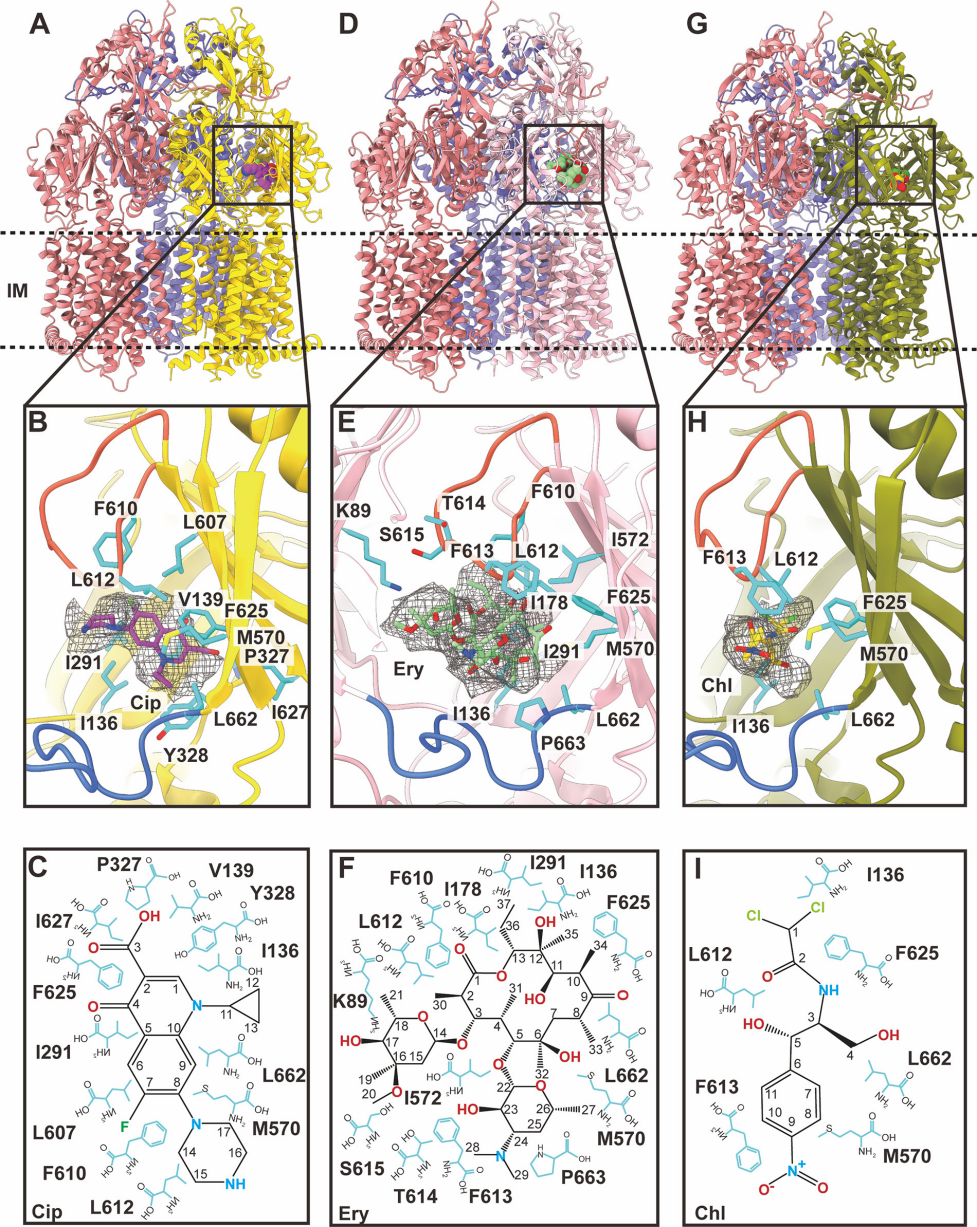
Cryo-EM structures of RE-CmeB-Cip, RE-CmeB-Ery, and RE-CmeB-Chl. (A) The ribbon diagram of the structure of the side view of the RE-CmeB-Cip trimer. The “access”, “binding”, and “extrusion” state protomers are colored in pink, slate, and yellow, respectively. The bound Cip molecule within the “binding” protomer is colored violet. (B) The Cip binding site. The residues that participate in Cip binding are shown with cyan sticks. The bound Cip molecule is colored violet. The cryo-EM density of Cip is colored gray. The F-loop and G-loop are colored blue and orange, respectively. (C) The schematic diagram of the Cip binding site. The amino acids that are important for Cip binding are shown as cyan sticks. (D) The ribbon diagram of the structure of the side view of the RE-CmeB-Ery trimer. The “access”, “binding”, and “extrusion” state protomers are colored in pink, slate, and tint, respectively. The bound Ery molecule within the “binding” protomer is colored green. (E) The Ery binding site. The residues that participate in Ery binding are in cyan sticks. The bound Ery molecule is colored green. The cryo-EM density of Ery is colored gray. The F-loop and G-loop are colored blue and orange, respectively. (F) The schematic diagram of the Ery binding site. The amino acids that are important for Ery binding are shown as cyan sticks. (G) The ribbon diagram of the structure of the side view of the RE-CmeB-Chl trimer. The “access”, “binding”, and “extrusion” state protomers are colored in pink, slate, and smudge, respectively. The bound Chl molecule within the “binding” protomer is colored yellow. (E) The Chl binding site. The residues that participate in Chl binding are shown with cyan sticks. The bound Chl molecule is colored yellow. The cryo-EM density of Chl is colored gray. The F-loop and G-loop are colored blue and orange, respectively. (F) The schematic diagram of the Chl binding site. The amino acids that are important for Chl binding are shown as cyan sticks.

**(i) Structure of RE-CmeB-Cip.** We incubated a 20 μM RE-CmeB-nanodisc sample with 1 mM Cip for 2 h on ice to form the RE-CmeB-Cip complex. Then, we collected single-particle cryo-electron microscopy (cryo-EM) images of this complex (Fig. S2). The reconstituted sample led to a cryo-EM map at a nominal resolution of 3.38 Å (Fig. 3A; Fig. S2; Table S1), thereby allowing us to obtain a structural model of the RE-CmeB-Cip complex at this resolution.

The overall structure of RE-CmeB-Cip is almost identical to that of the RE-CmeB-Amp trimer, with the three protomers displaying the “access”, “binding”, and “extrusion” forms, respectively (Fig. 3A; Table S2). The superimposition of the RE-CmeB-Cip and RE-CmeB-Amp trimers results in an overall root-mean-square-deviation (RMSD) of 0.6 Å. Cip was only observed to bind within the “binding” protomer of RE-CmeB, whereas no Cip molecules were found in the “access” and “extrusion” protomers of the trimer.

In the RE-CmeB-Cip complex structure, Cip is housed in the large cavity created by the distal multidrug-binding sites of the “binding” protomer of RE-CmeB, where the periplasmic cleft is completely open. Within 4 Å of bound Cip, the residues I136, V139, I291, P327, Y328, M570, L607, F610, L612, F625, I627, and L662 participate in anchoring this antibiotic (Fig. 3B and C). All 12 residues are hydrophobic in nature, suggesting that the binding is mostly governed by hydrophobic interactions. Surprisingly, no hydrogen bonds or significant electrostatic interactions are observed to facilitate the binding, highlighting the importance of these hydrophobic residues for substrate recognition.

**(ii) Structure of RE-CmeB-Ery.** We also incubated a 20 μM RE-CmeB-nanodisc sample with 1 mM Ery for 2 h on ice to form the RE-CmeB-Ery complex and determined this structure using single-particle cryo-EM (Fig. S3). The reconstituted sample led to a cryo-EM map at a nominal resolution of 3.39 Å (Fig. 3D; Fig. S3; Table S1), thereby allowing us to obtain a structural model of RE-CmeB-Ery. As with RE-CmeB-Cip, the three RE-CmeB protomers of the RE-CmeB-Ery complex exhibit the “access”, “binding”, and “extrusion” conformations, respectively (Fig. 3D; Table S2). The superimposition of the RE-CmeB-Ery and RE-CmeB-Cip trimers gives rise to an overall RMSD of 0.5 Å, suggesting that the structures of these two trimers are nearly identical. Again, Ery was only seen to bind within the “binding” protomer of RE-CmeB, whereas no Ery molecules were observed in the “access” and “extrusion” protomers.

In the “binding” protomer of RE-CmeB-Ery, Ery binds at a site that is distinct from, but partially overlaps with, the Cip binding site. It appears that the Ery molecule spans both the proximal and distal drug-binding sites. The oxacyclotetradecanyl ring of Ery is mostly housed within the distal drug-binding pocket, leaving its two hexopyranosyl rings to occupy the proximal drug-binding site. The amino acids within 4 Å of bound Ery include K89, I136, I178, I291, M570, I572, F610, L612, F613, T614, S615, F625, L662, and P663 (Fig. 3E and F). Most of these 14 residues are hydrophobic in nature, underscoring that drug recognition is mainly governed by hydrophobic interactions.

**(iii) Structure of RE-CmeB-Chl.** In addition to RE-CmeB-Cip and RE-CmeB-Ery, we assembled the RE-CmeB-Chl complex and solved its cryo-EM structure (Fig. S4). The reconstituted sample led to a cryo-EM map at a nominal resolution of 3.12 Å (Fig. 3G; Fig. S4; Table S1). Like RE-CmeB-Cip and RE-CmeB-Ery, the trimeric RE-CmeB-Chl complex is asymmetric in conformation and displays the “access”, “binding”, and “extrusion” protomer structures (Fig. 3G; Table S2). The pairwise superimpositions of the RE-CmeB-Cip, RE-CmeB-Ery, and RE-CmeB-Chl trimers give rise to RMSD values between 0.4 and 0.6 Å, suggesting that the structures of these three trimers are nearly identical.

As with Cip and Ery, the bound Chl molecule was only found within the “binding” protomer, leaving the multidrug binding sites of both the “access” and “extrusion” protomers unoccupied. The binding mode of Chl within the “binding” protomer is similar to that of Ery, where Chl occupies both the proximal and distal drug-binding pockets. However, it appears that RE-CmeB utilizes a different subset of residues to secure this drug. Within 4 Å of bound Chl, the residues I136, M570, L612, F613, F625, and L662 are engaged in anchoring this drug (Fig. 3H and I), whereas the residues M570, L612, F625, and L662 are used for Amp, Cip, Ery, and Chl binding. The prevalence of the hydrophobic residues indicates that hydrophobic interactions are the main driving force for fastening Chl binding, and this is similar to what was observed for the other RE-CmeB drug-bound structures.

### Mutation of the key amino acids involved in drug binding.

**FIG 4:**
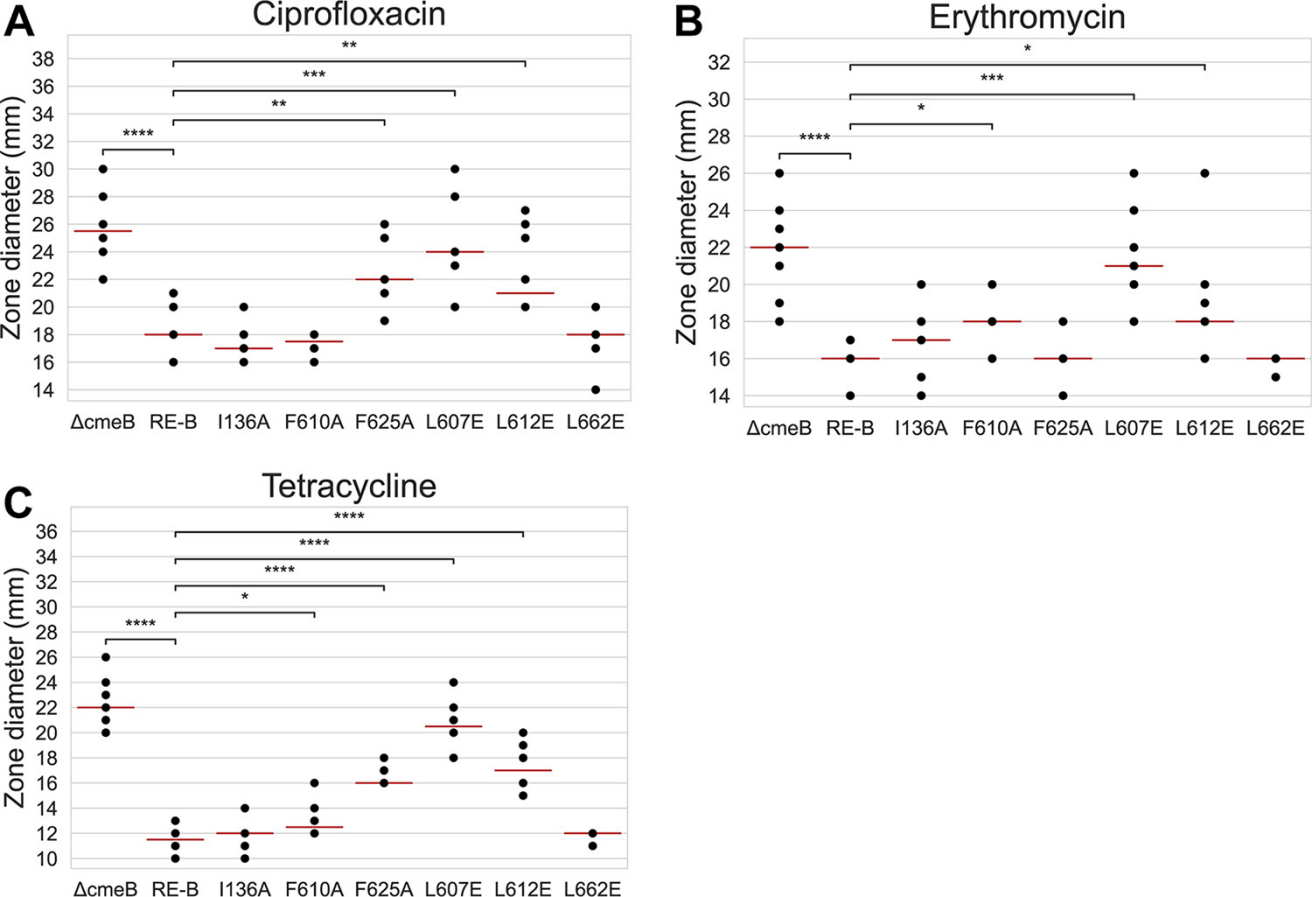
Susceptibility of various C. jejuni mutant constructs. Susceptibility to (A) Cip, (B) Ery, and (C) tetracycline, as determined via a disk diffusion assay. In each panel, the identity for each mutant construct is listed at the bottom. ΔcmeB, 11168Δ*cmeB::cat*; RE-B, 11168*cmeAB_RE_C*; and the other RE-CmeB mutants (I136A, F610A, F625A, L607E, L612E, and L662E). Each dot represents a single data point (zone diameter), and some are superimposed on one another. The red lines indicate the median for each strain. The differences between 11168*cmeAB_RE_C* and the other mutant strains were determined via Welch’s *t* test: *, *P < *0.05; **, *P < *0.01; ***, *P < *0.001; and ****, *P < *0.0001.

To verify the function of the identified key amino acid residues involved in antibiotic resistance, we generated I136A, F610A, F625A, L607E, L612E, and L662E mutant constructs of RE-CmeB in C. jejuni. These mutant strains, along with the *cmeB*-deleted mutant (11168 Δ*cmeB::cat*), were compared with a nonmutated RE-CmeB strain (11168*cmeAB_RE_C*) for their susceptibility to Cip, Ery, and tetracycline antibiotics by using a disc diffusion assay. As shown in Fig. 4, the zone diameters of inhibition of 11168Δ*cmeB::cat* were consistently larger than those of 11168*cmeAB_RE_C*, indicating that the loss of the CmeB transporter had a significant impact on antibiotic resistance. Compared to 11168*cmeAB_RE_C*, the F625A, L607E, and L612E mutations in RE-CmeB significantly increased the susceptibility of C. jejuni to Cip; the F610A, L607E, and L612E mutations increased the susceptibility to Ery; and the F610A, F625A, L607E, and L662E mutations increased the susceptibility to tetracycline (Fig. 4). Among the amino acid substitutions that were examined in this study, the L607E change increased the susceptibility to all three tested antibiotics and had the biggest impact, according to the zone diameters of inhibition, which were close to those of the CmeB-deletion mutant (11168Δ*cmeB::cat*). Interestingly, the I136A and L662E alterations did not affect the antibiotic susceptibilities in a significant manner. These results demonstrated the contributions of structurally important amino acids in mediating antibiotic resistance.

## DISCUSSION

The antibiotic-resistant Campylobacter represents a serious threat to public health (12, 13). One major mechanism that C. jejuni employs is the use of multidrug efflux pumps, which effectively lower intracellular drug concentrations well below toxic levels and simultaneously confer resistance to these antimicrobials. As the predominant antibiotic efflux system in C. jejuni, CmeABC functions cooperatively with other mechanisms (such as target mutations) to confer clinically relevant resistance to antibiotics. Sharing 81% amino acid homology with wild-type CmeB, the recently discovered RE-CmeB variant forms a unique branch phylogenetically and is functionally enhanced in antibiotic efflux, which results in elevated levels of resistance to multiple antibiotics, such as florfenicol, chloramphenicol, ciprofloxacin, erythromycin, and tetracycline (20). For example, a combination of the T86I mutation in GyrA with typical CmeABC gives rise to ciprofloxacin MICs of 8 to 32 μg/mL, whereas the same GyrA mutation combined with RE-CmeABC results in ciprofloxacin MICs of ≥256 μg/mL (20). The emergence and increased prevalence of RE-CmeB in Campylobacter is likely due to the antibiotic selection pressure that is imposed on this pathogen and makes the control of this infectious disease even more challenging, all of which underlines the need to decipher the structural basis and functional relationship of this important transporter.

To initiate structure-guided drug design to combat infections caused by C. jejuni, we solved the cryo-EM structures of the RE-CmeB multidrug efflux pump in the absence and presence of Cip, Ery, and Chl. The RE-CmeB pump relies on the PMF and functions via a drug/proton antiport mechanism. Coupled with the export of drug molecules toward the periplasm, protons are imported into the cytoplasm for energy coupling. Our cryo-EM maps unambiguously depict conformational differences of the side chains of residues D408, D409, K934, N935, and T972 within the transmembrane domain. These essential residues are conserved among the HAE-RND efflux pumps and constitute the proton-relay network. It has been found that a conserved lysine residue is critically important for proton transfer across the proton-relay network, where it participates as a proton sweeper to guide this process across the cytoplasmic membrane. We depicted distinct conformations of the K934 residue in different protomers within the RE-CmeB trimer, agreeing with what we previously observed in the N. gonorrhoeae MtrD (30, 31), A. baumannii AdeB (26, 32), and A. baumannii AdeJ (33, 34) multidrug efflux pumps.

As mentioned previously, the structures of the RE-CmeB and CmeB trimers are distinct from each other. A detailed inspection indicates that the major difference between these two structures is that the three protomers of the CmeB trimer display the “resting”, “binding”, and “extrusion” conformations, whereas those of the RE-CmeB trimer present the “access”, “binding”, and “extrusion” states. This difference in structure may not necessarily be related to the difference in the protein sequences of these two membrane proteins. They may simply reflect the fact that these protomers may need to go through various conformational states to complete the transport cycle by coupling with proton transfer in the proton-relay network.

Our cryo-EM data reveal details of the Cip, Ery, Chl, and Amp drug-binding sites of RE-CmeB. These four binding sites partially overlap each other. The phenomenon of manipulating slightly different residues to accommodate the binding of different drugs is often found in multidrug binding proteins, as these proteins often use different subsets of residues to accommodate the binding of compounds with distinct scaffolds. In RE-CmeB, this multidrug binding phenomenon is particularly pronounced. RE-CmeB utilizes slightly different residues in a large binding pocket to bind different drugs, thereby maximizing its capability to recognize a wide spectrum of distinct classes of antibiotics. Another observation based on the structural information is that RE-CmeB can coordinate both the proximal and distal sites and simultaneously use these two sites to recognize and anchor antibiotics. To bind these drugs, RE-CmeB often uses hydrophobic residues, such as I136, M570, L612, F625, and L662. Interestingly, F625 is a conserved residue, and this corresponding phenylalanine has been observed to be critical for drug recognition in E. coli AcrB (37), N. gonorrhoeae MtrD_CR103_ (30), and A. baumannii AdeJ (33, 34). Thus, hydrophobic interactions may be a general mechanism that governs drug recognition in RE-CmeB.

The manner by which RE-CmeB recognizes drugs seems to be distinct, compared to those of other multidrug efflux pumps within the HAE-RND family. For example, we previously observed that the N. gonorrhoeae MtrD_CR103_ multidrug efflux pump utilizes the distal-drug binding site to anchor the Ery drug. This drug is mainly bound by the MtrD_CR103_’s aromatic residues, such as F136, F176, Y325, F568, F610, F612, and F625, deep inside the pump (30). Our cryo-EM structure of RE-CmeB-Ery shows that the bound Ery molecule spans both the proximal and distal drug-binding sites. In addition to aromatic and hydrophobic residues, it was observed that some charged and polar residues, such as K89, T614, and S615, are involved in Ery binding. The fact that Ery is partially bound at the proximal and distal sites may indicate that there is no real boundary between these two sites. Thus, RE-CmeB can employ a large collection of residues from these two sites to optimize binding.

Previously, we resolved two cryo-EM structures of the A. baumannii AdeJ multidrug efflux pump that is bound by the tetracycline drugs eravacycline and TP-6076, respectively (33, 34). The structures indicated that these two drugs were bound deep inside the distal drug-binding site of AdeJ, where its aromatic residues, including F136, F178, F277, Y327, F611, F613, F616, F618, and F629, are actively engaged to secure the binding (33, 34). It is not known whether RE-CmeB uses a similar or distinct binding mode to recognize the tetracycline class of drugs. Given the fact that RE-CmeB utilizes a distinct binding mode to anchor Ery, Chl, and Amp, where these bound drugs partially occupy both the proximal and distal binding sites, it is possible that RE-CmeB may employ a different binding mode with a distinct set of residues to secure tetracycline binding, in comparison with that observed in AdeJ (33, 34). Further structural studies of the RE-CmeB pump with other antibiotics are needed to fully understand the detailed molecular mechanism that governs the multidrug recognition of this efflux pump.

Site-specific mutagenesis confirmed the roles of specific amino acids in antibiotic resistance (Fig. 4). These mutated amino acids were identified from a structural analysis as being important for drug binding. Mutations such as F610A, F625A, and L612E produced antibiotic-dependent changes in susceptibility, suggesting the variable role of F610, F625, and L612 in interacting with different antibiotics. However, the L607E mutation increased the susceptibility to all tested antibiotics, underlying the particular importance of this residue. Two mutations, namely, I136A and L662E, did not alter the susceptibility to the tested antibiotics, despite them being involved in antibiotic binding, suggesting that the individual mutation of I136 or L662 does not have much impact on the function of RE-CmeB. Given that the interaction with antibiotics is the collective effect of multiple amino acids, it is possible that the impacts of the I136A and L662E mutations on antibiotic susceptibility are incremental and that the disk diffusion assay may not be sensitive enough to detect the change. Thus, the simultaneous mutation of multiple amino acids involved in drug binding may be necessary to demonstrate the collective impact. Additionally, antibiotic resistance, as a phenotype, is affected not only by drug binding but also by the rate of entry into the periplasmic cleft. Therefore, the amino acids at the antibiotic entrance site may also be mutated for the evaluation of antibiotic susceptibility changes. All of these possibilities will be examined in future studies.

## MATERIALS AND METHODS

### Expression and purification of RE-CmeB.

The C. jejuni RE-CmeB multidrug efflux pump was cloned into the pET15bΩ*cmeB_RE_* expression vector in frame with a 6×His tag at the N terminus. The plasmid was transfected into E. coli BL21(DE3)Δ*acrB* cells, which harbor a deletion in the chromosomal *acrB* gene of E. coli, for the overproduction of the RE-CmeB membrane protein. The cells were grown in 12 L of LB medium supplemented with 100 μg/mL ampicillin at 37°C. When the OD_600 nm_ reached 0.5, the expression of RE-CmeB was induced with 0.2 mM isopropyl-β-d-thiogalactopyranoside (IPTG). The cells were then harvested within 4 h of induction. The collected bacterial cells were resuspended in low salt buffer (100 mM sodium phosphate [pH 7.2], 10% glycerol, 1 mM ethylenediaminetetraacetic acid (EDTA), and 1 mM phenylmethanesulfonyl fluoride [PMSF]) and disrupted with a French pressure cell. The membrane fraction was collected and washed twice with high salt buffer (20 mM sodium phosphate [pH 7.2], 2 M KCl, 10% glycerol, 1 mM EDTA and 1 mM PMSF) and once with final buffer (20 mM Na-HEPES [pH 7.5] and 1 mM PMSF). The membrane protein was then solubilized in 2% (wt/vol) n-dodecyl-β-d-maltoside (DDM). The insoluble material was removed via ultracentrifugation at 100,000 × *g*. The extracted protein was then purified using a Ni^2+^-affinity column. The purity of the RE-CmeB protein (>95%) was judged using SDS-PAGE stained with Coomassie Brilliant Blue. The purified protein was then dialyzed against 20 mM Na-HEPES (pH 7.5) and concentrated to 7 mg/mL (60 μM) in a buffer containing 20 mM Na-HEPES (pH 7.5) and 0.05% DDM.

### Nanodisc preparation.

To assemble RE-CmeB into nanodiscs, a mixture containing 20 μM RE-CmeB, 45 μM MSP (1E3D1), and 930 μM E. coli total extract lipid was incubated for 15 min at room temperature. 0.8 mg/mL prewashed Bio-beads (Bio-Rad) were added to remove the DDM detergent. The resultant mixture was incubated for 1 h on ice, and this was followed by overnight incubation at 4°C. The protein-nanodisc solution was filtered through 0.22 μm nitrocellulose-filter tubes to remove the Bio-beads. To separate the free nanodiscs from the RE-CmeB-loaded nanodiscs, the filtered protein-nanodisc solution was purified using a Superose 6 column (GE Healthcare) that was equilibrated with 20 mM Tris-HCl (pH 7.5) and 100 mM NaCl. Fractions corresponding to the size of the trimeric RE-CmeB-nanodisc complex were collected for cryo-EM sample preparation. 

### Cryo-EM sample preparation.

For imaging apo-RE-CmeB, a 20 μM RE-CmeB-nanodisc sample was directly applied to glow-discharged holey carbon grids (Quantifoil Cu R1.2/1.3, 300 mesh), blotted for 18 s, and then plunge-frozen in liquid ethane using a Vitrobot (Thermo Fisher). For imaging the RE-CmeB-Cip, RE-CmeB-Ery, or RE-CmeB-Chl, a 20 μM RE-CmeB-nanodisc sample was incubated with 1 mM Cip, Ery, or Chl for 2 h on ice to form the corresponding RE-CmeB-drug complexes. The samples were then applied to glow-discharged holey carbon grids (Quantifoil Cu R1.2/1.3, 300 mesh), blotted for 18 s, and then plunge-frozen in liquid ethane using a Vitrobot. All of the grids were then transferred into cartridges prior to data collection.

### Data collection.

For the apo-RE-CmeB sample, the images were collected in the super-resolution mode at 81 K magnification on a Titan Krios that was equipped with a K3 direct electron detector (Gatan). The physical pixel size was 1.07 Å/pix (super-resolution of 0.535 Å/pix). Each micrograph was exposed to a total dose of 36.1 e−/Å^2^ for 3.5 s, and 39 frames were captured using SerialEM (40). For the RE-CmeB-Cip, RE-CmeB-Ery, or RE-CmeB-Chl sample, each micrograph was collected over 38 frames, with a total dose of approximately 36 e−/Å^2^ over 3.5 s, using SerialEM (40).

### Data processing.

For apo-RE-CmeB, the super-resolution image stack was aligned and binned by 2, using patch motion. The contrast transfer function (CTF) was estimated using patch CTF in cryoSPARC (41). A procedure for the blob picker, which was followed by 2D classification, was applied to generate templates for automated template picking. Initially, 8,177,869 particles were selected after autopicking in cryoSPARC (41). Several iterative rounds of 2D classifications, which were followed by *ab initio* and heterogeneous 3D classifications, were performed to remove false picks and classes with unclear features, ice contamination, or carbon. A single round of nonuniform refinement that was followed by local refinement with nonuniform sampling resulted in 3.08 Å resolution cryo-EM maps for apo-RE-CmeB, based on the gold standard Fourier shell correlation (FSC 0.143) (Fig. S1).

For RE-CmeB-Amp, RE-CmeB-Cip, RE-CmeB-Ery, or RE-CmeB-Chl, the same procedure was used to generate templates for automated template picking. Initially, 8,177,869 particles for Amp, 1,206,616 particles for Cip, 1,738,896 particles for Ery, and 2,731,285 particles for Chl were selected after autopicking in cryoSPARC (41). Several iterative rounds of 2D classifications as well as *ab initio* and heterogeneous 3D classifications were performed to remove false picks and classes with unclear features. Nonuniform refinement that was followed by local refinement with nonuniform sampling resulted in 3.16 Å, 3.38 Å, 3.39 Å, and 3.12 Å resolution cryo-EM maps for RE-CmeB-Amp, RE-CmeB-Cip, RE-CmeB-Ery, and RE-CmeB-Chl, based on the gold standard Fourier shell correlation (FSC 0.143) (Fig. S3).

### Model building and refinement.

The building of the models was based on the cryo-EM maps, respectively. The CmeB structure (PDB ID: 5LQ3) (21) was used and fitted into the corresponding density maps using Chimera (42). The subsequent model rebuilding was performed using Coot (43). Structural refinements were performed using the phenix.real_space_refine program (44) from the PHENIX suite (45). The final atomic model was evaluated using MolProbity (46). The statistics that are associated with the data collection, 3D reconstruction, and model refinement are included in Table S1.

### Mutagenesis experiments.

To examine the roles of representative amino acids of RE-CmeB in antibiotic resistance, we first generated a *cmeB*-deleted (Δ*cmeB*) mutant by using C. jejuni 11168. A DNA fragment that contained the chloramphenicol resistance gene (*cat*) flanked by a 400 bp sequence on each side was synthesized (Gene Scripts, Piscataway, NJ). The flanking sequences corresponded to the immediate upstream and downstream sequences of *cmeB*. The synthetic DNA fragment was used as the donor DNA and was electroporated into C. jejuni 11168 by using a Bio-Rad GenePulser Xcell Electroporation System (Hercules, CA). Transformants were selected on Mueller-Hinton (MH) plates containing 15 μg/mL of chloramphenicol and were confirmed via PCR by using sequencing primers, which verified the insertion of the *cat* gene in place of *cmeB* with the concurrent deletion of *cmeB*, thereby resulting in the generation of the C. jejuni 11168 Δ*cmeB::cat* construct. This construct was used as the background strain for generating various mutants that were used in this study.

Then, we generated a plasmid template to facilitate the substitution of various amino acids in RE-CmeB. The *cmeABC* operon from C. jejuni 11168 was amplified via PCR by using the primers cmeABC-F and cmeABC-R (Table S3). The pUC18 plasmid of E. coli was linearized via inverse PCR with the primers pUC18-F/pUC18-R (Table S3). A NEB Gibson Assembly Kit (Ipswich, MA) was then utilized to ligate the two fragments and form plasmid pUC18Ω*cmeABC*. This plasmid was then transformed into E. coli DH5α (NEB, Ipswich, MA), and transformants were selected on LB media containing 100 μg/mL ampicillin. The plasmids were purified by using an alkaline lysis method, and the insertion of *cmeABC* into pUC18 was verified via restriction enzyme digestion.

Using pUC18Ω*cmeABC* as the template, an inverse PCR was conducted by using the primers LVcmeB-F/and LVcmeB-R (Table S3) to linearize the plasmid and delete *cmeB*. The primers cmeB-F and cmeB-R were used to amplify *RE-cmeB* from the genomic DNA of C. coli DH161 (20). Gibson assembly was then used to combine these two fragments to form a hybrid pUC18 construct with *cmeB* being replaced by *RE-cmeB* between *cmeA* and *CmeC*. This construct was transformed into E. coli DH5α, and transformants were selected on LB plates with 100 μg/mL ampicillin. The resulting plasmid was named pUC18Ω*cmeAB_RE_C* (*B_RE_* indicates *RE-cmeB*) and was sequenced to confirm the correct gene arrangement.

The pUC18Ω*cmeAB_RE_C* plasmid was used to generate various amino acid substitutions in RE-*cmeB*, These included the I136A, F610A, F625A, L607E, L612E, and L662E changes. The amino acid specific mutant constructs were generated by using a NEB Q5 Site-directed Mutagenesis Kit (Ipswich, MA) by following the instructions from the manufacturer. The mutagenic primers are listed in Table S3. For each mutagenesis experiment, 2 μL of the reaction mixture were added to E. coli DH5α cells that were supplied by the mutagenesis kit, and transformants were selected on LB plates with 100 μg/mL ampicillin. The plasmids were purified and subjected to Sanger sequencing to confirm that the desired amino acids substitutions were made in RE-CmeB.

The pUC18Ω*cmeAB_RE_C* plasmid and its derivatives with the site-specific changes in *RE-cmeB* were transformed into C. jejuni 11168Δ*cmeB*::*cat* via electroporation. For each transformation, 1 μg of the DNA of a purified plasmid was added to 11168Δ*cmeB*::*cat* competent cells, and selection was carried out on MH agar with either 0.2 μg/mL of tetracycline or 160 μg/mL of cholic acid. The concentrations of tetracycline and cholic acid that were used for the selection are above the MICs of the two compounds in C. jejuni 11168Δ*cmeB::cat* (15), which allowed for the selection of transformants that had obtained *RE-cmeB* (11168*cmeAB_RE_C*) or its derivatives. DNA sequencing was utilized to confirm that the *cat* gene was replaced by *RE-cmeB* and that its mutant derivatives between *cmeA* and *cmeC*, as well as the desired single amino acid mutations, were made in *RE-cmeB*.

### Disk diffusion assay.

The susceptibility of various C. jejuni mutant constructs to antibiotics were determined via a disk diffusion assay. Briefly, a MH agar plate was inoculated with a single C. jejuni strain (250 μL at an optical density of 1.0 OD_600_). After drying for approximately 5 to 10 min, blank diffusion disks (6 mm in diameter) (BD, Franklin Lakes, NJ) were placed onto the surface of the agar plate, with one disk in each quadrant. 10 μL of an antibiotic solution was added to each disk, and the total amount of antibiotic for each disk was 0.2 μg for tetracycline and 0.5 μg for Cip and Ery. The plates were incubated at 42°C under microaerophilic conditions for 24 h, and then the zone diameters of inhibition were recorded for the antibiotics. The experiment was repeated at least three times for each strain-antibiotic combination. The differences in zone diameters between 11168cmeAB_RE_C and the other mutants were analyzed via Welch’s *t* test.

### Data availability.

The atomic coordinates and EM maps of apo-RE-CmeB, RE-CmeB-Amp, RE-CmeB-Cip, RE-CmeB-Ery, and RE-CmeB-Chl have been deposited with the PDB accession codes 8GJJ, 8GJK, 8GJL, 8GK0, and 8GK4 and the EMDB accession codes EMD-40091, EMD-40092, EMD-40093, EMD-40177 and EMD-40179.
