# Supplementary material for: Cryo-Electron Microscopy Structures of a Campylobacter Multidrug Efflux Pump Reveal a Novel Mechanism of Drug Recognition and Resistance
Source: Microbiol Spectr. 2023 Jun 8;11(4):e01197-23. doi: 10.1128/spectrum.01197-23 (PMC10434076; doi:10.1128/spectrum.01197-23)
Supplement: Supplemental file 1 — Supplemental material. Download spectrum.01197-23-s0001.pdf, PDF file, 5.9 MB [file spectrum.01197-23-s0001.pdf]

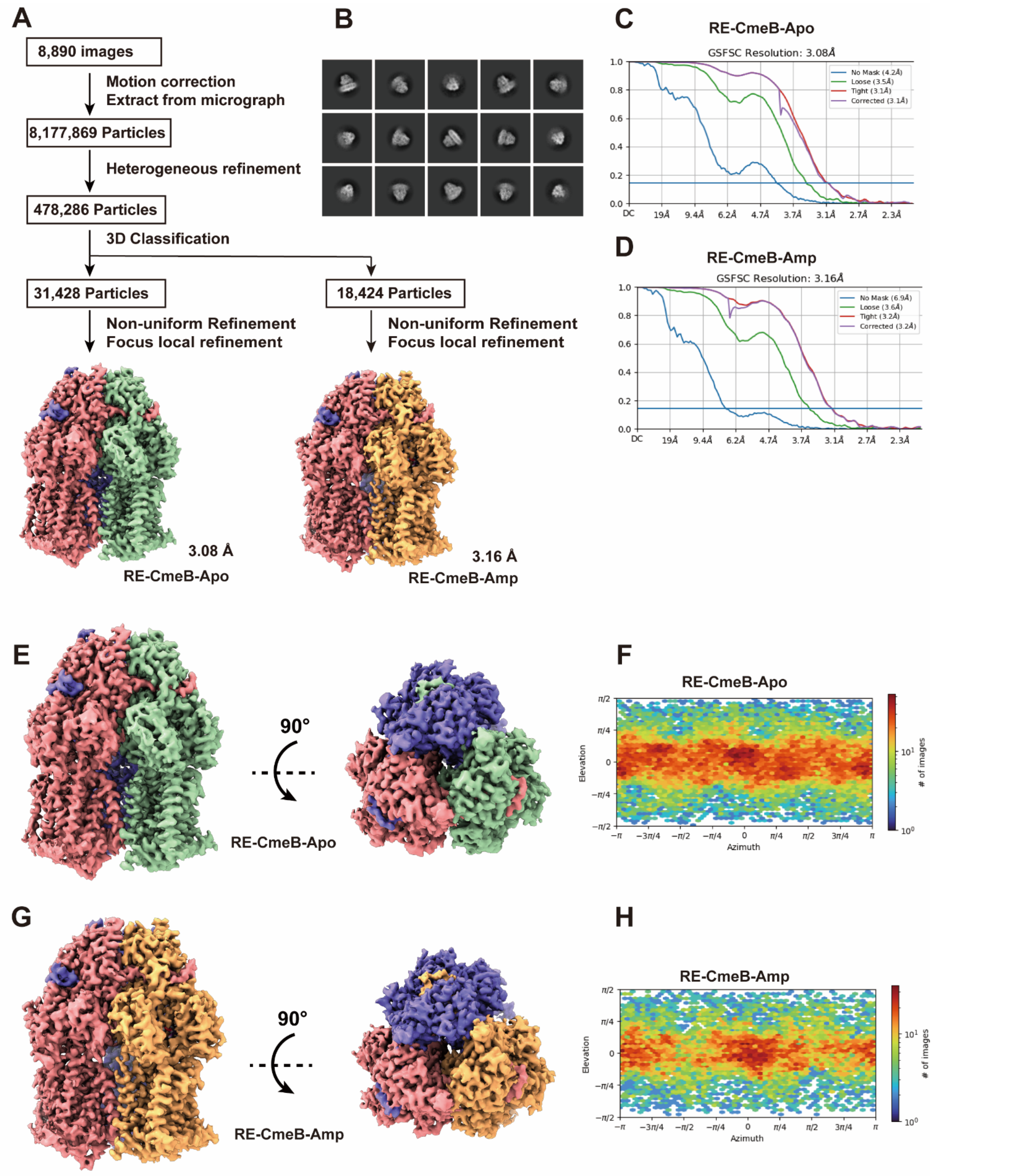

Fig S1. RE-CmeB Data processing. (A) Data processing workflow of RE-CmeB. (B) Representative 2D classes of RE-CmeB. (C-D) Gold-Standard Fourier shell correlation (GS-FSC) curves of RE-CmeB-Apo and RE-CmeB-Amp. (E) Side and top views of RE-CmeB-Apo density maps. (F) Direction distribution plot of RE-CmeB-Apo. (G) Side and top views of RE-CmeB-Amp density maps. (H) Direction distribution plot of RE-CmeB-Amp.

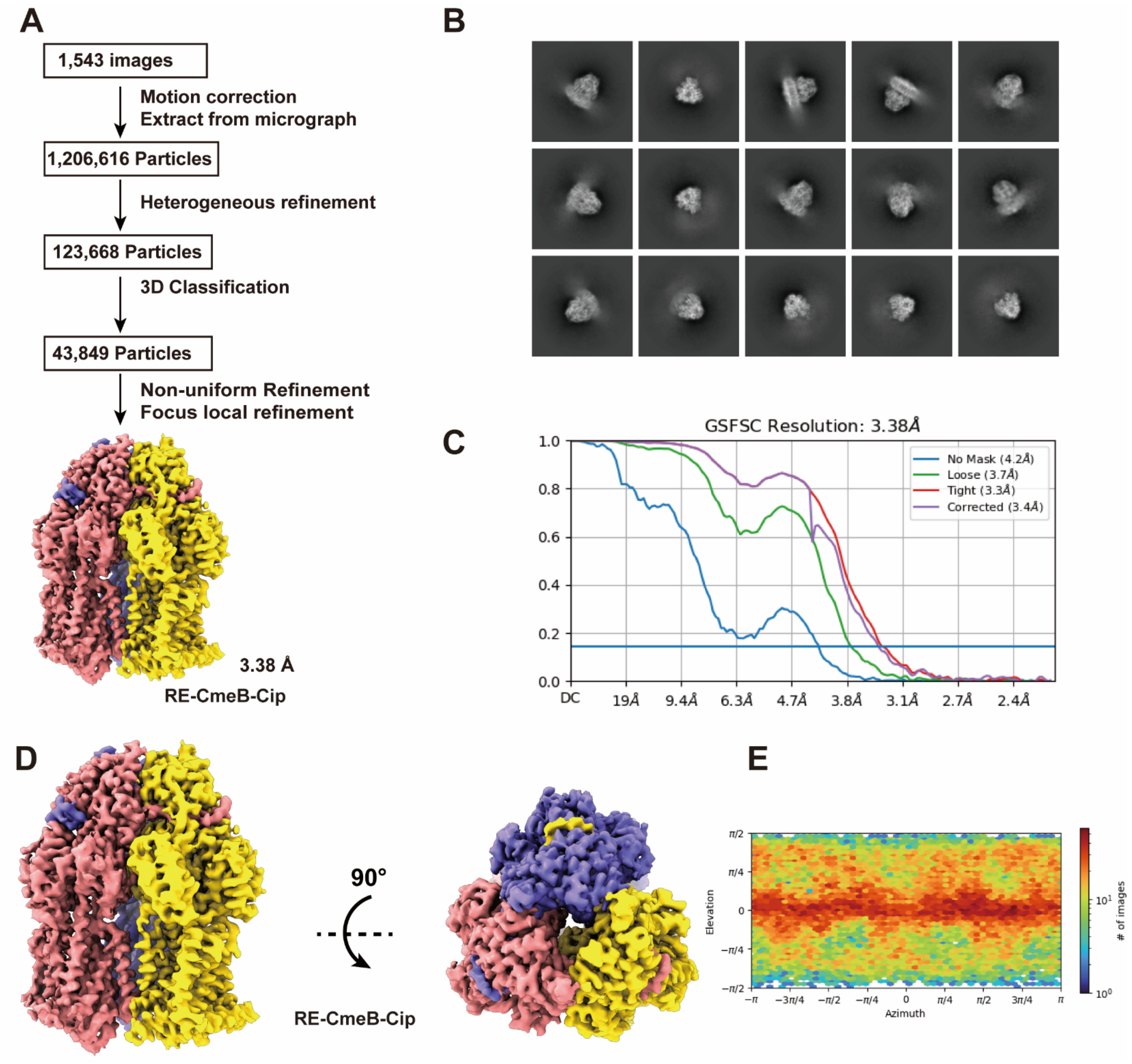

Fig S2. RE-CmeB-Cip Data processing. (A) Data processing workflow of RE-CmeB-Cip. (B) Representative 2D classes of RE-CmeB-Cip. (C) Gold-Standard Fourier shell correlation (GS-FSC) curves of RE-CmeB-Cip. (D) Side and top views of RE-CmeB-Cip density maps. (E) Direction distribution plot of RE-CmeB-Cip.

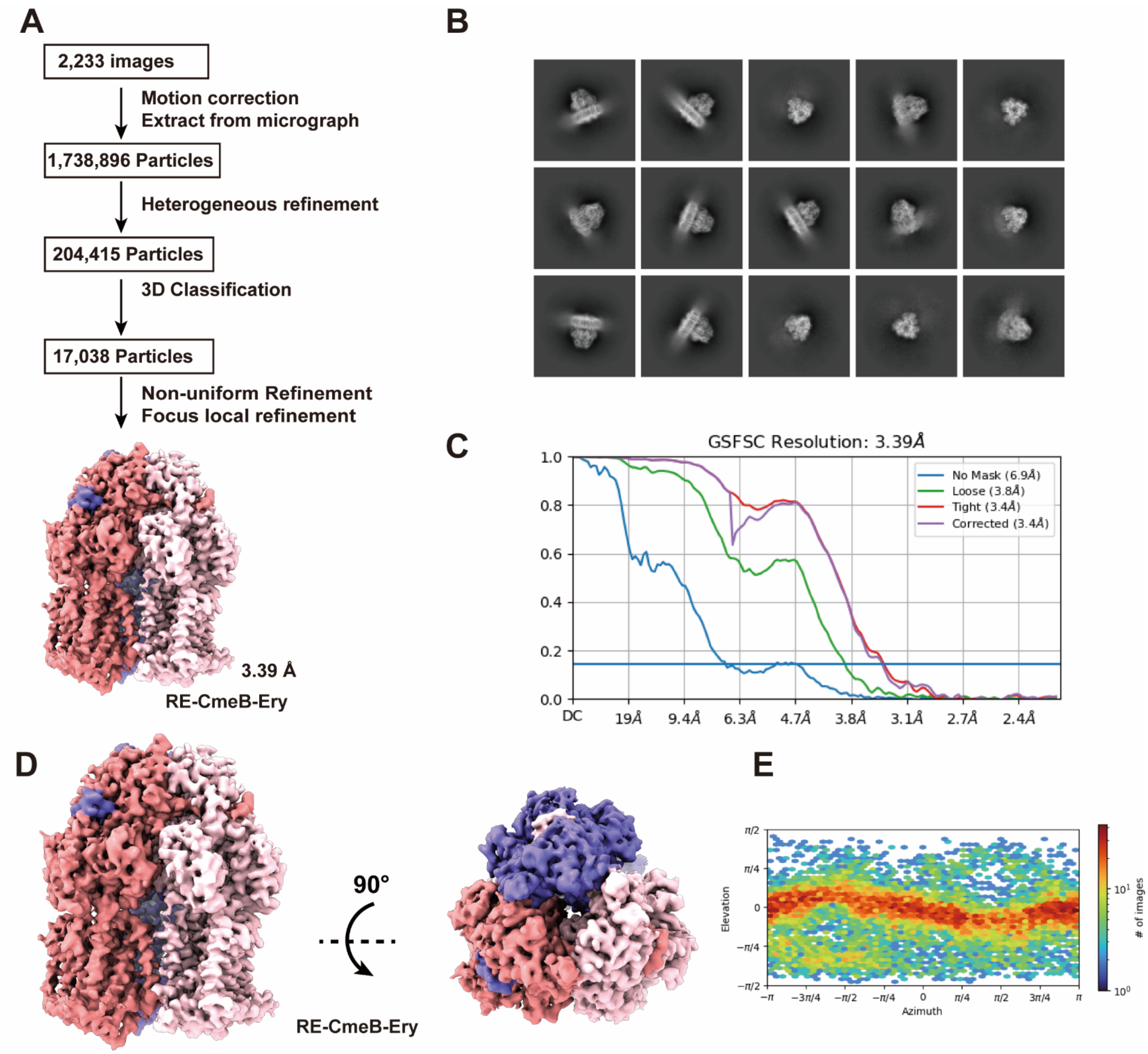

Fig S3. RE-CmeB-Ery Data processing. (A) Data processing workflow of RE-CmeB-Ery. (B) Representative 2D classes of RE-CmeB-Ery. (C) Gold-Standard Fourier shell correlation (GS-FSC) curves of RE-CmeB-Ery. (D) Side and top views of RE-CmeB-Ery density maps. (E) Direction distribution plot of RE-CmeB-Ery.

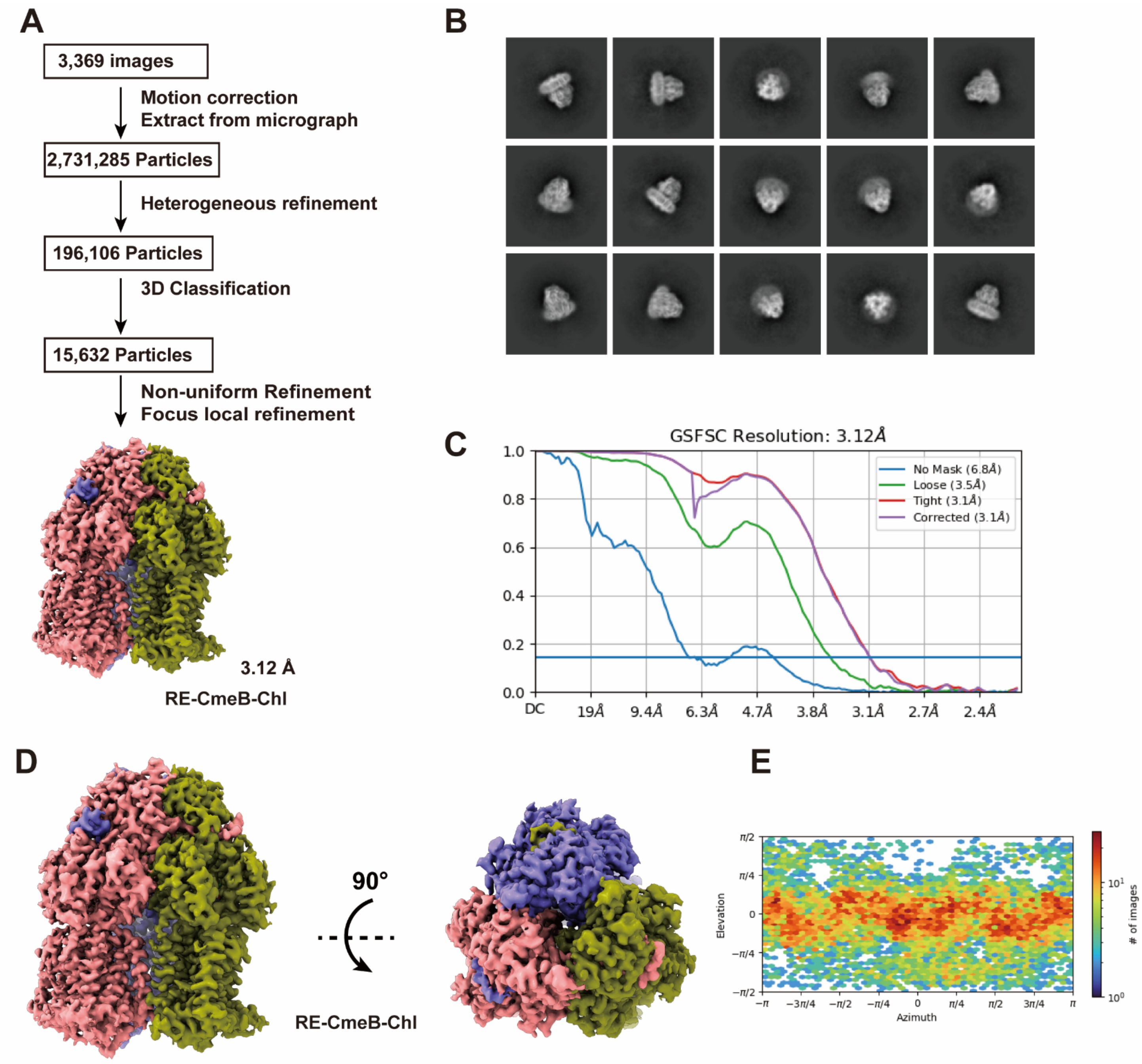

Fig S4. RE-CmeB-Chl Data processing. (A) Data processing workflow of RE-CmeB-Chl. (B) Representative 2D classes of RE-CmeB-Chl. (C) Gold-Standard Fourier shell correlation (GS-FSC) curves of RE-CmeB-Chl. (D) Side and top views of RE-CmeB-Chl density maps. (E) Direction distribution plot of RE-CmeB-Chl.

Table S1. RE-CmeB cryo-EM data collection and refinement statistics.

**Data Set**	**RE-CmeB-Apo**	**RE-CmeB-Amp**	**RE-CmeB-Cip**	**RE-CmeB-Ery**	**RE-CmeB-Chl**
**Data Collection and Processing**					
Magnification	81,000		81,000	81,000	81,000
Voltage (kV)	300		300	300	300
Electron Microscope	Krios-GIF-K3		Krios-GIF-K3	Krios-GIF-K3	Krios-GIF-K3
Defocus Range (µm)	-0.8 to -1.5		-0.8 to -1.5	-0.8 to -1.5	-0.8 to -1.5
Pixel Size (Å)	1.07		1.07	1.07	1.07
Total Dose ($e^-/Å^2$)	36.1		37.9	36.5	35.8
Number of Frames	39		38	38	38
Initial Micrographs	8,890		1,543	2,233	3,369
Initial Particles	8,177,869		1,206,616	1,738,896	2,731,285
Final Particles	31,428	18,424	43,849	17,038	15,632
GSFSC Resolution (Å)	3.08	3.16	3.38	3.39	3.12
**Refinement**					
Number of Protein Residues	3,096	3,096	3,096	3,089	3,094
Number of Ligands	0	1	1	1	1
**RMSD**					
Bond Lengths (Å)	0.005	0.008	0.004	0.005	0.007
Bond Angles (°)	0.572	1.376	0.684	0.806	1.280
**Validation**					
MolProbity Score	1.80	1.71	1.49	1.50	1.83
Clash Score	7.65	5.92	8.42	9.55	8.40
Rotamer Outliers (%)	2.74	2.87	0.65	0.31	2.37
**Ramachandran Plot (%)**					
Favored (%)	97.77	97.83	97.83	98.31	97.54
Disallowed (%)	0.00	0.03	0.00	0.00	0.03
**CC Score**					
Mask	0.84	0.82	0.83	0.84	0.77
Volume	0.82	0.80	0.82	0.83	0.76
Box	0.70	0.65	0.75	0.71	0.68

Table S2. Classification of RE-CmeB protomer states.

Protomer	Cleft State	Exit site distance, L125 to Y751	Hydrogen-bonded distance, K934 to				Protomer Assignment
			D408 (Å)	D409 (Å)	N935 (Å)	T972 (Å)	
RE-CmeB-Apo, A	Closed	13.99	-	-	2.81	3.03	Extrusion
RE-CmeB-Apo, B	Open	9.62	3.07	-	-	-	Access
RE-CmeB-Apo, C	Open	8.88	3.08	2.92	-	-	Binding
RE-CmeB-Amp, A	Closed	14.28	-	-	2.75	2.96	Extrusion
RE-CmeB-Amp, B	Open	9.38	2.80	-	-	-	Access
RE-CmeB-Amp, C	Open	9.59	2.94	3.07	-	-	Binding
RE-CmeB-Cip, A	Closed	11.12	-	-	2.97	3.04	Extrusion
RE-CmeB-Cip, B	Open	9.52	2.86	-	-	-	Access
RE-CmeB-Cip, C	Open	9.39	2.95	2.95	-	-	Binding
RE-CmeB-Ery, A	Closed	14.42	-	-	2.84	3.16	Extrusion
RE-CmeB-Ery, B	Open	9.48	3.03	-	-	-	Access
RE-CmeB-Ery, C	Open	9.66	-	2.99	-	-	Binding
RE-CmeB-Chl, A	Closed	15.06	-	-	2.85	3.16	Extrusion
RE-CmeB-Chl, B	Open	9.91	2.97	-	-	-	Access
RE-CmeB-Chl, C	Open	9.75	-	2.93	-	-	Binding

RE-CmeB protomers were defined using three criteria; state of the periplasmic cleft (open or closed), measurement of the exit site (distance between Cα atoms of L125 and Y751) and hydrogen bond distances of the PTC (K934 to D408, D409, N935 and T972). Using these results, protomers were assigned as either resting, access, binding and extrusion.

Table S3. Key PCR primers used in this study.

Primers	Sequence (5'-3')	Purpose
cmeABC-F	catgcctgcaggtcgacacaaagttctaaggtttttaataattcc	Amplification of *cmeABC* from 11168
cmeABC-R	gtacccggggatcctctagaaagcactcttataaaggataaaaatg	
puc18-F	cctttataagagtgctttctagaggatcccc	Linearization of pUC18 vector for *cmeABC* insertion
puc18-R	aaaccttagaactttgtgtcgacctgcaggcatg	
cmeB-F	ctaattgaaattattttattcatgaaccttac	Amplification of RE-*cmeB* from *C. coli* DH161
cmeB-R	gaaattggagcacaataatgttttc	
LVcmeB-F	aaaatttagaaaacattattgtgctccaatttc	Linearization of pUC18cmeABC for RE-*cmeB* insertion
LVcmeB-R	aaagaggtaaggttcatgaataaaataatttc	
I136A-F	tacttcaagtgtagtagaagatg	RE-*cmeB* I136A mutagenesis
I136A-R	gtctctgtatattctgaagatag	
F610A-F	aaataaatcagctcctataagagaaacgcttg	RE-*cmeB* F610A mutagenesis
F610A-R	acaagctctttaaaagaaaatg	
F625A-F	Aagtataaaagctactgctgcagcattttc	RE-*cmeB* F625A mutagenesis
F625A-R	aaagattggagtcaaagag	
L607E-F	caaatcctatctcagaaacgcttgag	RE-*cmeB* L607E mutagenesis
L607E-R	atttatttacaagctctttaaaag	
L612E-F	gcttgtaaattcatcaaatcctataagag	RE-*cmeB* L612E mutagenesis
L612E-R	tctttaaaagaaaatgctgc	
L662E-F	tattggtggctcatttaaaaaataacttag	RE-*cmeB* L662E mutagenesis
L662E-R	ccaggtcttagtttaacag	
